# Comparing Day 5 versus Day 6 euploid blastocyst in frozen embryo transfer and developing a predictive model for optimizing outcomes: a retrospective cohort study

**DOI:** 10.3389/fendo.2023.1302194

**Published:** 2024-01-04

**Authors:** Beining Yin, Sichen Li, Lin Sun, Zhiyi Yao, Yueyue Cui, Congli Zhang, Yile Zhang

**Affiliations:** ^1^ Reproductive Medicine Center, The First Affiliated Hospital of Zhengzhou University, Zhengzhou, Henan, China; ^2^ Henan Key Laboratory of Reproduction and Genetics, First Affiliated Hospital of Zhengzhou University, Zhengzhou, Henan, China

**Keywords:** preimplantation genetic testing, frozen embryo transfer, D5/D6 euploid blastocysts, blastocyst morphology, predictive model

## Abstract

**Background:**

Optimal protocols for frozen-thawed embryo transfer (FET) after preimplantation genetic testing (PGT) remain unclear. This study compared Day 5 (D5) and Day 6 (D6) blastocysts and evaluated predictors of FET success.

**Methods:**

A total of 870 patients with genetic diseases or chromosomal translocations who received PGT at the First Affiliated Hospital of Zhengzhou University from January 2015 to December 2019 were recruited. All patients underwent at least one year of follow-up. Patients were divided into groups according to the blastocyst development days and quality. Univariate and multivariate logistic regression were applied to identify risk factors that affect clinical outcomes and to construct a predictive nomogram model. Area under the curve (AUC) of the subject’s operating characteristic curve and GiViTI calibration belt were conducted to determine the discrimination and fit of the model.

**Results:**

D5 blastocysts, especially high-quality D5, resulted in significantly higher clinical pregnancy (58.4% vs 49.2%) and live birth rates (52.5% vs 45%) compared to D6. Multivariate regression demonstrated the number of blastocysts, endometrial preparation protocol, days of embryonic development and the quality of blastocysts independently affected live birth rates (*P<0.05*). A nomogram integrating these factors indicated favorable predictive accuracy (AUC=0.598) and fit (GiViTI, *P=0.192*).

**Conclusions:**

Transferring high-quality D5 euploid blastocysts after PGT maximizes pregnancy outcomes. Blastocyst quality, blastocyst development days, endometrial preparation protocols, and number of blastocysts, independently predicted outcomes. An individualized predictive model integrating these factors displayed favorable accuracy for counseling patients and optimizing clinical management.

## Introduction

Preimplantation Genetic Testing (PGT) is a revolutionary set of techniques employed in the field of assisted reproductive technology (ART) to assess the genetic health of embryos prior to their implantation in the uterus. Preimplantation genetic testing (PGT) is now routinely utilized to identify euploid embryos with standard chromosome copy numbers for transfer in IVF cycles. Transfer of euploid embryos after PGT has been conclusively demonstrated to improve implantation rates and live birth outcomes compared to untested embryo transfer (1). Vitrification has enabled exceptionally high post-warming survival rates (>95%), making frozen-thawed embryo transfer (FET) a vital component of IVF treatment (2). However, several patient and treatment related factors could impact the viability of warmed euploid blastocysts. Addressing these critical knowledge gaps could assist in improving clinical pregnancy and live birth rates after thawed euploid embryo transfer.

A significant determinant of embryo quality is the duration of *in vitro* culture before vitrification. Prolonged culture till the blastocyst stage on D5 or D6 allows for preferable selection of viable embryos with supreme implantation competence (3). However, extended *in vitro* culture might also negatively impact the developmental ability of embryos by exacerbating errors in gene expression, metabolism, and epigenetic modifications (4). This raises significant debate regarding whether D5 or D6 blastocysts provide superior frozen-thawed pregnancy outcomes after PGT. While several studies demonstrate comparable viability between D5 and D6 vitrified-warmed blastocysts (5, 6), other reports indicate higher success rates with transfer of D5 blastocysts compared to developmentally delayed D6 after cryopreservation (7, 8). Along with the duration of culture, the morphological grade is a significant predictor of blastocyst quality and competence (9). Evidence demonstrates that transfer of high-quality blastocysts classified as 3BB or higher is associated with significantly higher implantation, clinical pregnancy and live birth rates than poor-quality blastocysts (10). However, it is unclear whether extended *in vitro* culture could compensate for a reduced morphological grade regarding reproductive potential. Additional randomized controlled trials (RCT) are required to conclusively establish if D5 blastocyst transfer confers superior reproductive outcomes compared to D6 blastocyst after PGT and cryopreservation. Another critical determinant of success with frozen-thawed embryo transfer is endometrial receptivity. For endometrial preparation before FET, patients undergo either natural cycle (NC) monitoring or artificial hormone replacement therapy (HRT) (11). Evidence regarding which protocol provides optimal pregnancy outcomes remains contradictory (12).

In conclusion, multiple factors critically impact the implantation potential and reproductive outcomes of euploid blastocysts after cryopreservation and transfer in FET cycles. Addressing these research questions through well-designed studies can optimize clinical practice recommendations for PGT and FET. Developing and validating predictive models based on critical determinants of cryopreserved blastocyst potential is also essential for individualized prognosis (13). Results from such investigations can assist in patient counselling and evidence-based clinical decision-making, improving overall outcomes with frozen embryo transfer.

## Materials and methods

### Study design and population

This was a retrospective cohort follow-up study. The clinical data of patients with genetic diseases or chromosomal translocations receiving PGT at the First Affiliated Hospital of Zhengzhou University from January 2015 to December 2019 were analyzed. A total of 870 first single frozen-thawed euploid blastocyst transfer cycles were recruited. All patients were followed up for at least one year.

**Figure 1:**
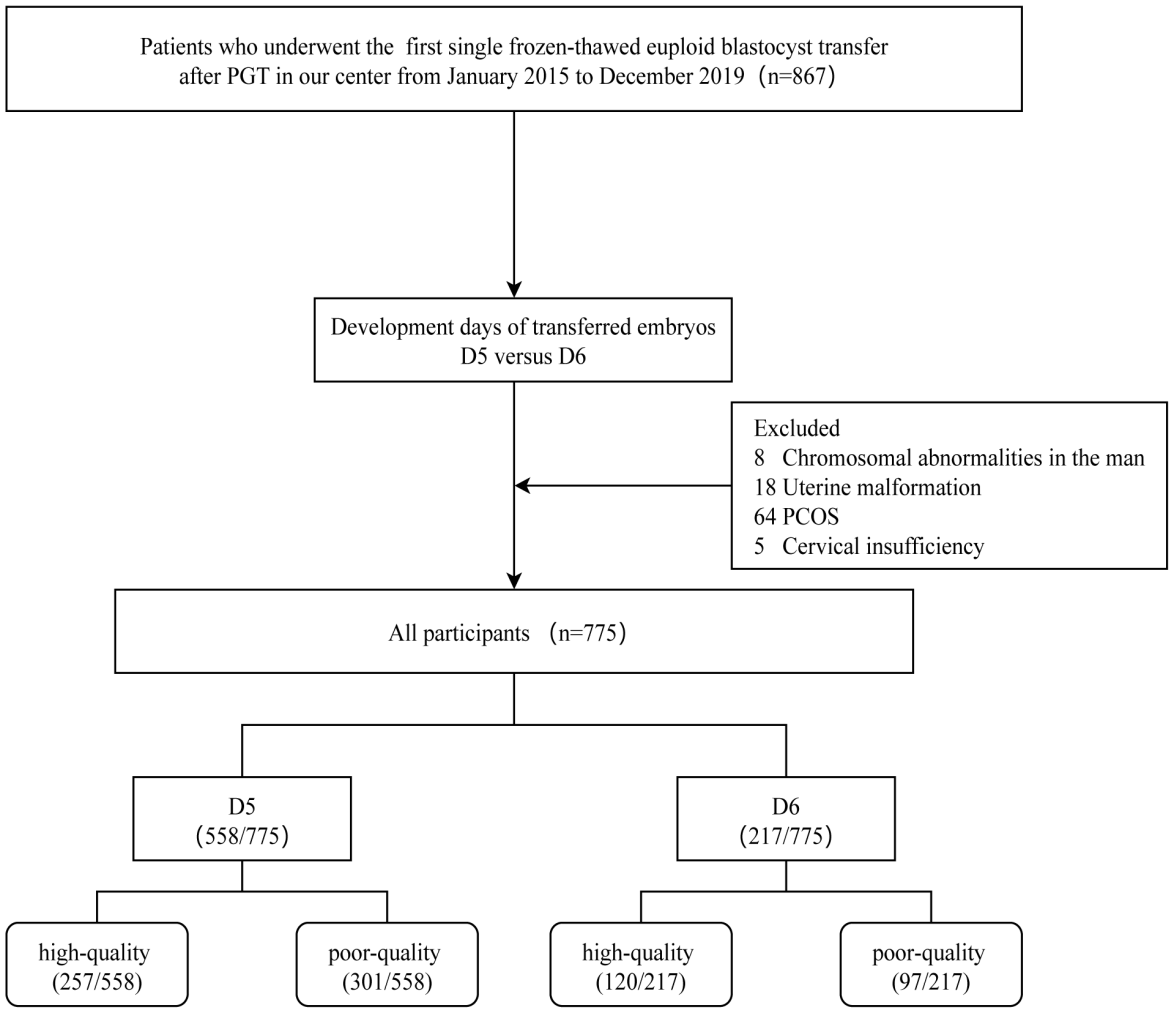
The flowchart of participants. PGT, Preimplantation genetic testing; PCOS, PolyCystic Ovary Syndrome.

The inclusion criteria for this study are as follows: 1) Patients who underwent their first single embryo thawed transfer at our reproductive center; 2) Patients who underwent preimplantation genetic testing (PGT); 3) Patients who underwent endometrium preparation using either a natural cycle or hormone replacement therapy (HRT) protocol, and the exclusion criteria: 1) Male chromosomal abnormalities; 2) Endometriosis; 3) Polycystic ovary syndrome (PCOS); 4) Cervical insufficiency; 5) Inner membrane thickness on conversion day < 7mm (14); 6) Uterine adhesions and malformations; 7) Patients with autoimmune infertility; 8) Patients with significant missing data. Of these, 95 were excluded from the study for the following reasons: male chromosomal abnormalities (n=8), endometriosis (n=18), poly cystic ovary syndrome (n=64), and cervical insufficiency (n=5). After screening, 775 eligible participants were included in the study (**Figure 1**).

Patients’ clinical data were obtained from the Clinical Reproductive Medical Record Cohort Database (CCRM/EMRCD) of the Reproductive Medical Center of The First Affiliated Hospital of Zhengzhou University, and follow-up data were obtained from the results of the telephone follow-up or from the obstetric medical record system of our hospital. This study was approved by the Institutional Review Board and Ethics Committee of the First Affiliated Hospital of Zhengzhou University (reference number: 2020-KY-256). The information of the statistical recipients had been anonymized and follows the ethical principles of the Declaration of Helsinki. Due to the retrospective study, the patient did not sign the informed consent form.

### Morphological evaluation of the blastocyst

Methods of ovarian stimulation, transvaginal ultrasound egg retrieval, IVF/ICSI (Intracytoplasmic sperm injection), embryo culture, embryo vitrification, and thawing of embryos are described in previous publications by researchers at our center (15). The egg retrieval day was defined as D0. On the morning of the fifth or sixth day after egg retrieval, experienced embryologists scored the blastocysts according to the Gardner and Schoolcraft scoring systems (16). First, the blastocysts were classified into different stages according to the degree of expansion. The quality of the inner cell mass (ICM)/trophectoderm (TE) of the stage 3 to 6 blastocysts was further assessed. If both ICM and TE scores are above grade B (3BB), this blastocyst is defined as a high-quality blastocyst; otherwise, it is considered a poor-quality blastocyst.

### Blastocyst biopsy and euploidy diagnosis

PGT encompasses several specialized techniques. Each technique provides unique insights into different aspects of genetic health assessment. The PGT signal detection techniques used by our reproductive center mainly include Next Generation Sequencing, Single Nucleotide Polymerism, and Karyomapping. In general, embryos with mosaic proportions below 30% are considered suitable for transfer.

Next-Generation Sequencing (NGS) is a high-throughput genetic sequencing technology widely employed in the field of reproductive medicine. It involves fragmenting DNA samples into millions of small segments, followed by parallel sequencing and reassembly to obtain comprehensive genomic or exomic information. NGS enables simultaneous detection of numerous genetic regions, providing high-resolution genetic data. In the context of Preimplantation Genetic Testing (PGT), NGS finds extensive applications, including PGT-A for detecting chromosomal aneuploidies, PGT-M for identifying specific monogenic mutations, and PGT-SR for assessing structural rearrangements such as inversions, translocations, or segmental deletions in embryos.

Single Nucleotide Polymorphism (SNP) is a common form of genetic variation characterized by single-nucleotide substitutions within the genome. SNP analysis involves the detection of SNP marker sites within embryo cell samples, facilitating the determination of genetic information and genotypes in specific chromosomal regions.

Karyomapping is an advanced PGT technique that integrates SNP analysis to simultaneously detect both chromosomal abnormalities and specific gene mutations. By analyzing SNP marker sites within embryo cells, Karyomapping provides information about the genetic content and genotypes in different chromosomal regions.

### Endometrial preparation protocols

Natural Cycle (NC): On days 8-10 of the menstrual cycle, transvaginal ultrasound is performed to monitor follicular growth and endometrial thickness. When the dominant follicle reaches a diameter of 14 mm, luteinizing hormone (LH) levels are monitored in urine both in the morning and evening. The day of the LH peak or the day of ovulation confirmed by ultrasound is considered the conversion day (D1). From the conversion day, vaginal progesterone capsules (Utrogestan, 100mg/capsule, Cyndea Pharma, S.L.) are inserted at a daily dose of 400mg. Starting from day 3 (D3), oral administration of dydrogesterone tablets (Duphaston, 10mg/tablet, Abbott Healthcare Products B.V) begins at a daily dose of 30mg. Embryo transfer is performed on day 5 (D5) following the standard protocol of our center, guided by abdominal ultrasound. After the procedure, vaginal progesterone gel (Crinone, 90mg/applicator, MERCK SERONO LIMITED.) at a daily dose of 90mg or vaginal progesterone capsules at a dose of 400mg/day are used. Oral administration of dydrogesterone tablets at a dose of 30mg/day continues until peripheral blood beta human chorionic gonadotropin (β-hCG) is tested after 14 days post-transfer to determine biochemical pregnancy. Abdominal ultrasound examination is performed on day 35 post-transfer to determine clinical pregnancy. If pregnancy is confirmed, progesterone support continues until day 45 post-transfer, after which vaginal progesterone gel or vaginal progesterone capsules are discontinued. Dydrogesterone tablets are discontinued on day 65 post-transfer.

Hormone Replacement Therapy (HRT): Starting from days 2-3 of the menstrual cycle, serum hormone levels are monitored. In the absence of abnormalities, estradiol valerate tablets (Progynova, 1mg/tablet, DELPHARM Lille S.A.S.) are taken at a daily dose of 4mg. The dosage is adjusted based on serum hormone levels and endometrial growth. On days 12-14 of medication, when the endometrial thickness reaches ≥ 7 mm, intramuscular injection of 60mg progesterone (Progesterone, 20mg/injection, Zhejiang Xianju Pharmaceutical Co., Ltd.) is added to induce endometrial transformation. The following day, oral administration of dydrogesterone tablets begins at a dose of 10mg/day, which is changed to 30mg/day after 3 days. Embryo transfer is performed according to the standard protocol of our center, guided by abdominal ultrasound, on the sixth day of progesterone injection. After the procedure, vaginal progesterone gel at a daily dose of 90mg or vaginal progesterone capsules at a dose of 400mg/day are used. Oral administration of dydrogesterone tablets at a dose of 30mg/day continues until peripheral blood β-hCG is tested after 14 days post-transfer to determine biochemical pregnancy. Abdominal ultrasound examination is performed on day 35 post-transfer to determine clinical pregnancy. If pregnancy is confirmed, progesterone support continues until day 45 post-transfer, after which vaginal progesterone gel or vaginal progesterone capsules are discontinued. Dydrogesterone tablets are discontinued on day 65 post-transfer.

### Group and observation indicators

Based on the development day of the transplanted blastocysts, all patients were divided into groups D5 (n=558) and D6 (n=217). According to the blastocyst quality, the patients continued to be divided into high-quality D5 group (n=257), poor-quality D5 group (n=301), high-quality D6 group (n=120) and poor-quality D6 group (n=97). Baseline characteristics in both groups were prospective during the visit. The following data were collected: age (years), height (m), body weight (kg), infertility type, BMI, essential endocrine FSH, E2, AMH, AFC and infertility causes (e.g., ovulation disorders, male factors, fallopian tube factors, premature ovarian failure (POF) or idiopathic infertility). The primary clinical outcome was obtained from the consensus reached by the American Society of Reproductive Medicine in 2017 (17). Clinical pregnancy was defined as one or more gestational cysts detected by ultrasound. Live birth was defined as the delivery of at least one live infant after 22 weeks of gestation.

### Statistical analysis

All statistical analyses were conducted in R statistical environment (R version 4.1.3). The quantitative data with variance meeting the normal distribution and homogeneity were tested by one-way analysis of variance test, and the results were expressed as mean ± standard deviation; the data not meeting the normal distribution and variance heterogeneity were tested by Kruskal-Wallis test, and the results were expressed as median (Q1, Q3). Qualitative data were compared between multiple groups using the chi-square test. Factors with statistically significant differences in the univariate analysis results were subsequently included in the multivariate logistic regression model, and the risk factors ultimately included in the model were selected using the forward stepwise method.

Furthermore, based on the regression coefficients of the independent variables, we used the *RMS package* for nomogram drawing. We built an individualized nomogram prediction model to predict patients’ clinical outcomes based on risk factors. The discrimination of the model prediction results is usually evaluated by calculating the area under the curve (AUC) of the subject’s operating characteristic curve. The AUC values are between 0.5 and 1.0. The closer the AUC value is to 1, the better the discriminative power of the predictive model is. Generally, a prediction model with an AUC of 0.5-0.75 is considered acceptable.

Subsequently, the GiViTI calibration band was applied to test the goodness of fit of the predictive model (18). Unlike the conventional Hosmer-Lemeshow goodness-of-fit test, the GiViTI calibration band aims to reveal the relationship between predicted and observed probabilities by fitting polynomial logistic functions and indicates the direction, degree, and risk grade affected by these deviations. A statistically significant deviation from the bisector occurs when the 95% CI boundaries of the GiViTI calibration belt do not encompass the bisector (the ideal line of perfect calibration). The significant P-value in the calibration test indicates insufficient evidence that the model was a poor fit. GiViTI Calibration bands were drawn using the *givitiR package*. Two-sided *P <0.05* were considered statistically significant.

## Results

### Comparison of differences between groups

**Table 1** presented patients’ baseline characteristics and clinical outcomes in groups D5 and D6. The data exhibited that the two groups were comparable for baseline characteristics, such as female age, duration and type of infertility, BMI and basal hormone levels. The clinical pregnancy and live birth rates in the D5 group were 54.84% and 48.92%, significantly higher than 44.24% and 39.17% in the D6 group (*P <0.05*).

**Table 1:** Baseline characteristics and pregnancy outcomes of patients.

	Overall	D5	D6	P value
PGT cycles	775	558	217	
Female age at oocyte retrieval	29.726 (4.094)	29.778 (4.146)	29.594 (3.963)	0.576
Female age at blastocyst transfer	30.063 (4.104)	30.120 (4.139)	29.917 (4.016)	0.537
Age group (%)				0.249
>35	78 (10.06)	61 (10.93)	17 (7.83)	
≤35	697 (89.94)	497 (89.07)	200 (92.17)	
Infertility years	2.000 [1.000, 3.000]	2.000 [1.000, 3.000]	2.000 [1.000, 3.000]	0.358
Pregnancy numbers	1.588 (1.505)	1.613 (1.536)	1.525 (1.424)	0.468
Abortion Numbers	1.000 [0.000, 2.000]	1.000 [0.000, 2.000]	1.000 [0.000, 2.000]	0.799
Infertility type (%)				0.472
Primary	244 (31.48)	171 (30.65)	73 (33.64)	
Secondary	531 (68.52)	387 (69.35)	144 (66.36)	
BMI (kg/m2)	22.786 (2.974)	22.759 (2.932)	22.854 (3.088)	0.691
BMI group(%)				0.472
>24	244 (31.48)	171 (30.65)	73 (33.64)	
≤24	531 (68.52)	387 (69.35)	144 (66.36)	
Basic endocrine				
FSH(mIU/mL)	6.390 [5.300, 7.300]	6.480 [5.350, 7.320]	6.240 [5.188, 7.228]	0.167
E2(pg/mL)	35.620 [25.400, 48.430]	35.510 [25.000, 47.790]	36.330 [26.135, 51.912]	0.627
AMH(ng/mL)	3.640 [2.280, 5.870]	3.670 [2.317, 5.615]	3.610 [2.210, 6.040]	0.931
Endometrial preparation protocol (%)				0.670
Hormone replacement therapy	592 (76.39)	429 (76.88)	163 (75.12)	
Natural cycle	183 (23.61)	129 (23.12)	54 (24.88)	
AFC	16.130 (5.783)	16.005 (5.678)	16.451 (6.047)	0.341
AFC group(%)				0.231
≤10	152 (19.61)	103 (18.46)	49 (22.58)	
>10	623 (80.39)	455 (81.54)	168 (77.42)	
No.of retrieved oocytes	18.000 [12.000, 23.000]	17.000 [12.250, 23.000]	18.000 [12.000, 23.000]	0.496
Endometrial Thickness(mm)	11.000 [10.000, 13.000]	11.000 [10.000, 13.000]	11.000 [9.000, 13.000]	0.470
Clinical pregnancy rate(%)	402 (51.87)	306 (54.84)	96 (44.24)	0.010
Live birth rate(%)	358 (46.19)	273 (48.92)	85 (39.17)	0.018

PGT, Preimplantation genetic testing; BMI, body mass index; FSH, follicle-stimulating hormone; E2, estradiol; AMH, antimullerian hormone; AFC, antral follicle count.

AMH, antimullerian hormone; AFC, antral follicle count.

**Table 2** presented each group’s baseline characteristics and clinical outcomes when the patients were further grouped according to the transplanted blastocyst quality. In the whole population, the clinical pregnancy rate and live birth rate were 51.87% and 46.19%, while in the high-quality D5 group, this data reached 58.37% and 52.53%, which was significantly higher than the other three groups (*P <0.001*). Subsequently, the analysis of the poor-quality D5 group and high-quality D6 group exhibited no significant differences between some baseline characteristics and clinical outcomes (**Supplementary Table 1**). However, it is worth noting that the total number of AFC in the poor-quality D5 group were significantly higher than those in the high-quality D6 group (*P <0.05*).

**Table 2:** Baseline characteristics and pregnancy outcomes of patients.

	High-quality D5 group	Poor-quality D5 group	High-quality D6 group	Poor-quality D6 group	Pvalue
PGT cycles	257	301	120	97	
Female age at oocyte retrieval	30.113 (4.332)	29.492 (3.965)	29.792 (4.317)	29.351 (3.482)	0.248
Female age at blastocyst transfer	30.420 (4.279)	29.864 (4.006)	30.167 (4.424)	29.608 (3.445)	0.270
Age group (%)					0.251
>35	33 (12.84)	28 (9.30)	11 (9.17)	6 (6.19)	
≤35	224 (87.16)	273 (90.70)	109 (90.83)	91 (93.81)	
Infertility years	2.000 [1.000, 3.000]	2.000 [1.000, 3.000]	2.000 [1.000, 3.000]	2.000 [1.000, 4.000]	0.676
Pregnancy numbers	1.716 (1.635)	1.525 (1.443)	1.625 (1.567)	1.402 (1.222)	0.269
Abortion Numbers	1.000 [0.000, 2.000]	1.000 [0.000, 2.000]	1.000 [0.000, 2.000]	1.000 [0.000, 2.000]	0.732
Infertility type (%)					0.701
Primary	74 (28.79)	97 (32.23)	40 (33.33)	33 (34.02)	
Secondary	183 (71.21)	204 (67.77)	80 (66.67)	64 (65.98)	
BMI (kg/m^2^)	22.678 (2.996)	22.828 (2.879)	22.932 (3.359)	22.756 (2.726)	0.874
BMI group(%)					0.333
>24	78 (30.35)	93 (30.90)	46 (38.33)	27 (27.84)	
≤24	179 (69.65)	208 (69.10)	74 (61.67)	70 (72.16)	
Basic endocrine					
FSH(mIU/mL)	6.505 [5.405, 7.340]	6.460 [5.230, 7.290]	6.250 [5.320, 7.400]	6.050 [5.175, 7.035]	0.392
E2(pg/mL)	34.000 [24.280, 46.530]	36.450 [26.778, 49.365]	38.325 [25.372, 48.745]	35.040 [27.085, 57.098]	0.380
AMH(ng/mL)	3.460 [2.125, 5.630]	3.890 [2.590, 5.490]	3.230 [2.070, 5.535]	4.170 [2.530, 6.762]	0.062
Endometrial preparation protocol					0.880
HRT	195 (75.88)	234 (77.74)	89 (74.17)	74 (76.29)	
NC	62 (24.12)	67 (22.26)	31 (25.83)	23 (23.71)	
AFC	15.992 (5.818)	16.017 (5.567)	15.940 (6.165)	17.073 (5.873)	0.402
AFC group(%)					0.090
≤10	54 (21.01)	49 (16.28)	32 (26.67)	17 (17.53)	
>10	203 (78.99)	252 (83.72)	88 (73.33)	80 (82.47)	
No.of retrieved oocytes	17.000 [12.000, 24.000]	18.000 [13.000, 22.000]	17.000 [11.000, 22.000]	19.000 [15.000, 26.000]	0.083
Endometrial Thickness(mm)	11.000 [10.000, 13.000]	11.000 [10.000, 13.000]	11.000 [10.000, 13.000]	11.000 [9.000, 13.000]	0.644
Clinical pregnancy rate(%)	150 (58.37)	156 (51.83)	59 (49.17)	37 (38.14)	0.007
Live birth rate(%)	135 (52.53)	138 (45.85)	54 (45.00)	31 (31.96)	0.007

PGT, Preimplantation genetic testing; BMI, body mass index; NC, natural cycle; HRT, hormone replacement therapy; FSH, follicle-stimulating hormone; E2, estradiol;

AMH, antimullerian hormone; AFC, antral follicle count.

### Risk factors affecting pregnancy outcomes

Univariate logistic regression was conducted to dissect the effect of each variable on pregnancy outcome (**Table 3** and **Supplementary Table 2**). The results demonstrated that the quality of the transplanted blastocysts was positively correlated with the live birth rate, and the days of embryonic development (D5 and D6) were negatively correlated with the clinical pregnancy rate and live birth rate. Other statistically significant risk factors included the endometrial preparation protocol and the number of blastocyst embryos formed (*P <0.05*). Furthermore, no subsequent analysis was performed since only a single risk factor influenced the clinical pregnancy rate. The inclusion of risk factors selected from the univariate analysis into the unconditional binary multivariable Logit model (**Table 3** and **Supplementary Table 2**) displayed that the number of blastocysts (OR,1.039; 95% CI, 1.003-1.076*; P*=0.031), endometrial preparation protocol (OR,1.462; 95% CI, 1.044-2.047; *P*=0.027), days of embryonic development (OR,0.659; 95% CI, 0.476-0.911; *P*=0.012), and the quality of blastocysts (OR,1.453; 95% CI, 1.087-1.940; *P*=0.012) were independent risk factors affecting the live birth rate. It was suggested that adopting a natural cycle for intimal preparation and avoiding transplanting D6 blastocysts and poor-quality blastocysts are beneficial to improving the live birth rate. Meanwhile, the forming blastocyst number also exhibited a potential clinical predictive value. The collinear diagnostic analysis of the above independent risk factors also demonstrated that there was no multicollinearity between them.

**Table 3:** Univariate and multivariate logistic regression analysis on the effect of live birth rate.

	Univariate analysis		Multivariate analysis	
	OR (95% CI)	P value	OR (95% CI)	P value
Female age at oocyte retrieval	0.970 (0.937-1.005)	0.088	–	–
Female age at blastocyst transfer	0.971 (0.938-1.005)	0.094	–	–
Age group (>35 VS ≤35)	0.839 (0.523-1.347)	0.468	–	–
Pregnancy numbers	1.048 (0.954-1.151)	0.330	–	–
Abortion Numbers	1.035 (0.929-1.154)	0.532	–	–
Infertility years	0.953 (0.898-1.011)	0.109	–	–
Infertility type (Primary VS Secondary)	1.057 (0.780-1.432)	0.723	–	–
BMI	1.011 (0.964-1.060)	0.666	–	–
BMI group (>24 VS ≤24)	1.042 (0.769-1.413)	0.791	–	–
FSH	0.999 (0.996-1.003)	0.709	–	–
E2	1.000 (1.000-1.000)	0.990	–	–
AMH	1.013 (0.964-1.066)	0.606	–	–
Endometrial preparation protocol (NC VS HRT)	1.430 (1.026-1.995)	0.035	1.462 (1.044-2.047)	0.027
AFC	1.013 (0.988-1.038)	0.311	–	–
No. of retrieved oocytes	0.995 (0.977-1.012)	0.556	–	–
No. of formatted blastocysts	1.035 (1.000-1.071)	0.048	1.039 (1.003-1.076)	0.031
Endometrial Thickness(mm)	1.034 (0.978-1.092)	0.243	–	–
Blastocyst development day (Day 6 VS Day 5)	0.672 (0.489-0.925)	0.015	0.659 (0.476-0.911)	0.012
Blastocyst quality (High-quality VS Poor-quality)	1.362 (1.026-1.808)	0.032	1.453 (1.087-1.940)	0.012

BMI, body mass index; FSH, follicle-stimulating hormone; E2, estradiol; AMH, anti-mullerian hormone; NC, natural cycle;

HRT, hormone replacement therapy; AFC, antral follicle count.

### Construction and evaluation of the prediction models

**Figure 2:**
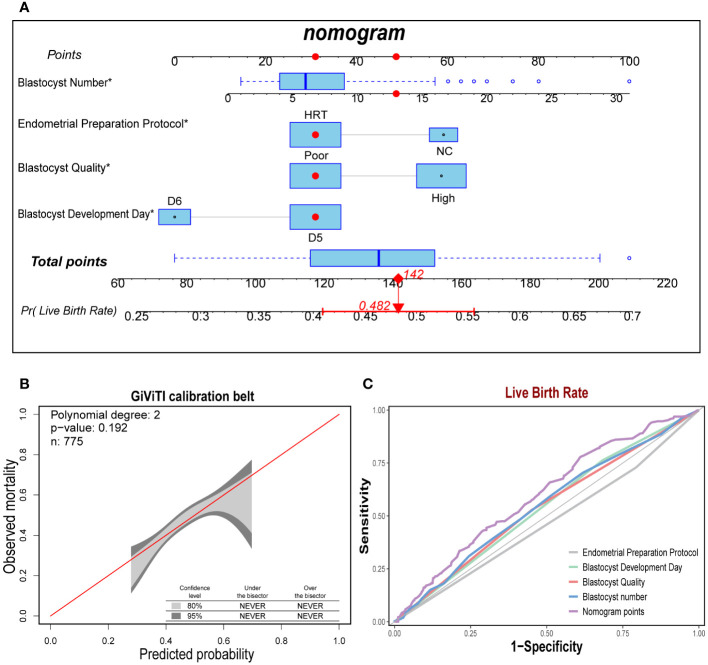
Construction and test of the prediction model for live birth rate. **(A)**. The nomogram exhibited four characteristics of a patient (Blastocyst Number = 14, Endometrial Preparation Protocol = HRT, Blastocyst Quality = Poor, Blastocyst Development Day = D6), with a total score of 142 points, and the predicted probability of live birth was 48.2%. **(B)**. The sample number of the prediction model was 775, and the diagonal bisector was within the 95% confidence interval (*P = 0.192*). **(C)**. The area under the curve of Norman score was 0.598 (95% CI: 0.559-0.638), exhibiting a relatively good discrimination degree.

Based on the four independent predictors of blastocyst number, endometrial preparation protocol, days of blastocyst development, and blastocyst quality, we fitted the live yield prediction model and constructed a personalized nomogram. Based on the nomogram, the total score was obtained by adding the scores corresponding to each predictor, and the probability value corresponding to the vertical line of the total score was the live birth rate predicted by the model (**Figure 2A**). Furthermore, the diagonal bisector was located within the 95% confidence interval of the GiViTI calibration band with no deviations and a GiViTI calibration test P-value of 0.192 (**Figure 2B**). It indicated that the model’s prediction was not statistically significant overprediction, and the model cannot be considered poorly fit. The predicted and actual probabilities of the model were strongly consistent. Further drawing the ROC curves of the prediction model displayed that the AUC of the model was 0.598 (95%CI: 0.559-0.638), which showed a relatively good discrimination degree, and the prediction model was considered acceptable (**Figure 2C**).

## Discussion

This retrospective cohort study analyzed pregnancy outcomes of 775 patients undergoing frozen-thawed blastocyst transfer after PGT. It evaluated the effects of blastocyst development time and quality on clinical pregnancy and live birth rates. The results demonstrated that transfer of D5 blastocysts, incredibly high-quality D5 blastocysts, significantly increased pregnancy and live birth rates compared to D6 blastocysts. Multivariate regression analysis revealed that blastocyst number, endometrial preparation protocol, blastocyst development time and quality were independent predictors of live birth rate. A predictive nomogram constructed using these factors displayed preferable discriminatory and calibration abilities.

The optimal timing for frozen embryo transfer has remained a controversial issue. During preimplantation development, the embryo undergoes complex molecular and cellular changes to gain developmental competence and prolonged *in vitro* culture might negatively impact embryonic viability (19). Some studies have compared outcomes of D3 embryo and D5/D6 blastocyst transfers, but results remain contradictory (20, 21). A randomized controlled trial found significantly higher pregnancy rates with D5 blastocyst transfer than D3 cleavage-stage embryos (3), indicating that culturing embryos to the blastocyst stage allows better selection of embryos with higher implantation potential.

However, evidence regarding differences between D5 and D6 blastocyst transfer is complicated. Taylor et al. retrospectively compared outcomes of single embryo transfer of 1508 D5 and 361 D6 blastocysts. They found no significant differences in aneuploidy rates, pregnancy or live birth rates between D5 and D6 blastocysts (5). Liebermann et al. compared vitrified D5 and D6 blastocysts to conventional slow freezing and found higher survival and pregnancy rates with D5 and D6 vitrification compared to slow freezing. However, pregnancy rates were comparable between vitrified D5 and D6 blastocysts at 52% and 39%, with no significant difference statistically (6). This suggested that D5 and D6 blastocysts might have similar reproductive potential. In contrast, several studies reported significantly higher implantation, clinical pregnancy and live birth rates with D5 frozen-thawed blastocysts than developmentally delayed D6 blastocysts (7). Our results were consistent with the latter study, demonstrating markedly higher clinical pregnancy and live birth rates after D5 blastocyst transfer compared to D6. A recent systematic review and meta-analysis examining factors influencing the implantation of haploid embryos revealed a significantly lower survival rate for single haploid embryo transfer (SET) on Days 6-7 compared to Day 5 embryos (OR, 1.49; 95% CI, 1.25-1.76; *P<0.001*) (22). These findings further supported our research conclusion that Day 5 blastocysts might demonstrate more preeminent viability outcomes compared to Day 6 blastocysts. Overall, most evidence indicated that post-PGT transfer of euploid D5 blastocysts maximized the chances of pregnancy success. However, reasonably successful outcomes could still be achieved with D6 blastocysts in some situations. This indicated a need for further RCT to provide more robust evidence favoring prioritized D5 embryo transfer. In summary, prolonged culture to the blastocyst stage appears beneficial, but whether D5 or D6 blastocysts provided optimal results requires further investigation.

Blastocyst morphology was an important indicator of its viability. The inner cell mass generated fetal tissues, while the trophectoderm formed the critical extraembryonic tissues for implantation and placental development (23). Algorithms combining morpho kinetic parameters with standard morphological criteria have been shown to predict blastocyst implantation success more accurate (24, 25). Several studies demonstrated higher implantation, clinical pregnancy and live birth rates after transferring high-quality blastocysts compared to euploid and aneuploid embryos (26, 27). Fragouli et al. found higher implantation and lower miscarriage rates with morphologically higher-graded blastocysts in studying the relationship between embryo morphology and developmental potential (28). Similarly, Irani et al. found comparable results when studying the impact of morphological grading of euploid blastocysts on implantation and ongoing pregnancy rates (10). Our results were unanimous with these findings, showing significantly higher pregnancy and live birth rates after transferring high-quality D5 blastocysts than poor-quality D5 and D6 blastocysts.

However, few studies directly compare poor-quality D5 blastocysts to high-quality D6 blastocysts. Our study found no statistically significant difference in pregnancy outcomes between these groups, suggesting even poor-quality D5 blastocysts might have similar developmental potential as high-quality D6 blastocysts. As far as we know, the relatively slower development and poorer viability of D6 blastocysts might explain this discrepancy (29, 30). Additionally, the shorter culture time of low-quality D5 blastocysts might synchronize their developmental stage with the receptive endometrium, whereas prolonged *in vitro* culture for high-quality D6 embryos could lead to displacement and increased risk of embryo asynchrony, stress, and compromised viability, potentially affecting successful implantation (7, 31).

Furthermore, Hashimoto et al. discovered that delayed growth of human blastocysts increases spindle abnormalities and reduces post-vitrification implantation potential (32). This abnormality might contribute to the similar implantation potential observed between high-quality D6 and low-quality D5 blastocysts. Prolonged *in vitro* culture can exacerbate errors in gene expression, epigenetic modifications, and mitochondrial activity, further impairing embryonic competence (33). Studies have demonstrated that the down regulation of oxidative phosphorylation genes, influenced by mitochondrial RNA, can affect oocyte quality, including fertilization and subsequent embryonic development (34). Additionally, accumulation of mtDNA mutations, decreased copy number, and reduced expression associated with mitochondrial defects can impact embryonic development (35), indicating that even high-quality blastocysts may accumulate a significant number of mtRNA mutations due to extended culture time, which can affect further development after implantation. Moreover, timely degradation of maternal RNA during the transition from the maternal to the zygotic genome has been demonstrated as crucial. Inhibiting its degradation leads to a mixed developmental state and embryo developmental failure (36, 37). Overall, considering the combination of genetic and epigenetic composition, developmental time, and physical conditions of the embryo, these factors collectively contributed to similar pregnancy outcomes observed in low-quality D5 blastocysts and high-quality D6 blastocysts.

Endometrial receptivity was another critical factor impacting pregnancy success. Under the influence of ovarian steroid hormones, the endometrium undergoes complex molecular changes to achieve a state receptive to embryo attachment, adhesion and implantation (38). The natural cycle relied on endogenous hormones for optimal secretory transformation of the endometrium, while HRT utilized exogenous steroids. A few evidence based on systematic reviews and meta-analyses indicates more favorable pregnancy outcomes with natural cycles than HRT (39, 40). This was consistent with our findings of higher live birth rates with natural cycle preparation. However, further research was warranted to elucidate the precise mechanisms of endometrial receptivity.

The advantages of this study were as follows. Firstly, all implanted blastocysts in this study underwent PGT, minimizing the impact of aneuploidy on pregnancy outcomes. Besides, our nomogram integrated the number of blastocysts formed, endometrial preparation method, blastocyst culture time and quality to provide an individualized prediction of live birth probability after frozen embryo transfer. While these factors have been identified previously, constructing and validating a robust predictive model represents a novel contribution. Given the rapid expansion of PGT, the nomogram could serve as a valuable counselling tool to manage patient expectations and guide clinical decision-making. Patients strongly favor personalized risk estimation rather than population averages to make informed treatment choices. Providing individualized outcome prediction based on patient and treatment characteristics is also aligned with the goals of precision medicine (41).

There are some limitations in this study. Firstly, being a retrospective survey, it was prone to inherent biases. Conducting prospective randomized controlled trials would provide better insights into the impact of prolonged *in vitro* culture and blastocyst quality on frozen embryo transfer outcomes. Additionally, the mechanisms underlying the higher implantation rates and improved pregnancy outcomes of D5 blastocysts compared to D6 blastocysts after PGT remain incompletely understood. Further experimental results are crucial to support the conclusions of this study, particularly in the comparison of low-quality D5 blastocysts with high-quality D6 blastocysts. Secondly, external validation of our cohort is necessary before implementing our nomogram in clinical practice. Other potential predictive factors, such as ploidy status, previous failed transfers, and freezing methods, have not been taken into consideration. Moreover, although live birth was selected as the primary outcome, neonatal outcomes were not evaluated. Follow-up studies assessing perinatal outcomes are essential. Incorporating additional predictive factors could enhance the discriminative ability and clinical utility of the model.

## Conclusion

In summary, this study demonstrated that post-PGT transfer of euploid D5 blastocysts maximized chances of pregnancy and live birth compared to D6 embryos. Blastocyst quality, blastocyst development days, endometrial preparation, and number of blastocysts available also impacted success rates. The predictive model provided individualized assessment to counsel patients, select embryos, and optimize clinical management. Further refinement and validation of the nomogram will support broader clinical application to guide treatment decisions and improve outcomes of frozen embryo transfer cycles.

## Data availability statement

The raw data supporting the conclusions of this article will be made available by the authors, without undue reservation.

## Ethics statement

The studies involving humans were approved by Ethics Committee of Scientific Research and Clinical Trials of the First Affiliated Hospital of Zhengzhou University. The studies were conducted in accordance with the local legislation and institutional requirements. Written informed consent for participation was not required from the participants or the participants’ legal guardians/next of kin because This study was a retrospective study.

## Author contributions

YZ: Conceptualization, Data curation, Resources, Supervision, Writing – review & editing. BY: Data curation, Formal analysis, Software, Writing – original draft. SL: Data curation, Visualization, Writing – original draft. LS: Writing – review & editing. ZY: Writing – review & editing. YC: Writing – review & editing. CZ: Writing – review & editing.

## Supplementary material

Supplementary Table 1 Baseline characteristics and pregnancy outcomes of poor-quality D5 group and high-quality D6 group.

Supplementary Table 2 Univariate and Multivariate logistic regression analysis on the effect of Clinical pregnancy rate.
